# A Novel RSSI Prediction Using Imperialist Competition Algorithm (ICA), Radial Basis Function (RBF) and Firefly Algorithm (FFA) in Wireless Networks

**DOI:** 10.1371/journal.pone.0151355

**Published:** 2016-07-20

**Authors:** Shidrokh Goudarzi, Wan Haslina Hassan, Aisha-Hassan Abdalla Hashim, Seyed Ahmad Soleymani, Mohammad Hossein Anisi, Omar M. Zakaria

**Affiliations:** 1 Communication System and Network (iKohza) Research Group, Malaysia-Japan International Institute of Technology (MJIIT), Universiti Teknologi Malaysia, Jalan Semarak, Kuala Lumpur 54100, Malaysia; 2 Faculty of Electrical and Computer Engineering International Islamic University Malaysia Kuala Lumpur, Kuala Lumpur, Malaysia; 3 Faculty of Computing, Universiti Teknologi Malaysia, UTM Johor Bahru, Johor 81310, Malaysia; 4 Department of Computer System and Technology, Faculty of Computer Science and Information Technology, University of Malaya, Kuala Lumpur 50603, Malaysia; Southwest University, CHINA

## Abstract

This study aims to design a vertical handover prediction method to minimize unnecessary handovers for a mobile node (MN) during the vertical handover process. This relies on a novel method for the prediction of a received signal strength indicator (RSSI) referred to as IRBF-FFA, which is designed by utilizing the imperialist competition algorithm (ICA) to train the radial basis function (RBF), and by hybridizing with the firefly algorithm (FFA) to predict the optimal solution. The prediction accuracy of the proposed IRBF–FFA model was validated by comparing it to support vector machines (SVMs) and multilayer perceptron (MLP) models. In order to assess the model’s performance, we measured the coefficient of determination (*R*^2^), correlation coefficient (*r*), root mean square error (*RMSE*) and mean absolute percentage error (*MAPE*). The achieved results indicate that the IRBF–FFA model provides more precise predictions compared to different ANNs, namely, support vector machines (SVMs) and multilayer perceptron (MLP). The performance of the proposed model is analyzed through simulated and real-time RSSI measurements. The results also suggest that the IRBF–FFA model can be applied as an efficient technique for the accurate prediction of vertical handover.

## Introduction

In recent decades, we have witnessed the astonishing development in wireless applications, devices and networks. In heterogeneous wireless networks, the (Vertical Handover) VH is an important factor in the provision of seamless mobility between varied network environments and an essential feature of all next-generation all-IP mobile network endeavors. In the context of future wireless networks, many analyses, studies and tutorials have been proposed in the literature [1–4]. These algorithms were classified into different groups based on the decision technique expended. Rakovic and Gavrilovska [5] proposed a novel method for Radio Access Technology (RAT) selection, namely, the Hopfield neural network RAT selection Mechanism (HRM), that utilized the Hopfield neural networks as a strong decision making tool. A new approach using information about data rate, monetary cost and received signal strength as different parameters to make a handover decision has been reported by [6]. The main weaknesses were exactly linked to the computation of the error function and Jacobian inversion for acquiring a matrix in which the dimensions were equal to the total of all the weights in the neural network. Hence, the requirement for memory was very high [7,8]. Existing algorithms [9] considered service fee, Received Signal Strength Information (RSSI), and user preference, etc. The proposed algorithm compared to the traditional RSSI based algorithm, enhanced outcomes significantly for both user and network as a consequence of the proposed fuzzy based handover systems [9]. In terms of hybrid categories, Nan et al. [10] proposed a PSO-FNN-based vertical handover decision algorithm that could make a reasonable handover decision intelligently based on the study of network status. Liu and Jiang [11] reported a novel vertical handover decision algorithm built on fuzzy logic with the assistance of grey theory and dynamic weights adaptation. A neuro-fuzzy multi-parameter-based Vertical Handover Decision Algorithm (VHDA) was proposed by [12] where the results of performance evaluation, carried out by a handover quality indicator (used to quantify QoS) which is related upon the ‘Ping-Pong’ effect, ESA and throughput, proved that the proposed VHDA offered better QoS than existing vertical handover methods. Pahlavan et al. (2000) was a good representation of the application of a fuzzy logic-based normalized quantitative decision algorithm and a differential prediction algorithm that had good accuracy.

With the possibility of network prediction, which has posed as a significant challenge in next-generation access networks, the usage of networks associated with low costs and high data rates can be maximized. In this regard, several methods have been proposed based on handover prediction [13]. Due to necessity of accurate and reliable VHO decisions, artificial and computational intelligence methods have been exhaustively used to predict vertical handover in numerous studies in the literature. Neural networks have been successfully applied to solve complicated problems by automatically learning the system’s behavior. The field of neural networks has applied in handover related issues in order to estimate signal decay[14], and also to predict users’ profiles[15,16].

In Received Signal Strength Indicator (RSSI)-based prediction algorithms, RSSI of the current attachment point is compared to the RSSI of the other available networks for the prediction of handover. Becvar et al.[2] suggest a handover mechanism to maximize the handover prediction efficiency using the parameter of the (RSSI)[17,18]. The authors have described two thresholds for RSSI for optimal use. Using a similar method in [19,20], a predictive RSS scheme with a Markov Decision Process (MDP) was proposed based on network selection for vertical handovers in heterogeneous wireless networks. In the first stage, a polynomial regression based methodology is used to predict whether a mobile node (MN) handover is nearer to or farther away from a wireless network. In the next stage, the candidate access network with the lowermost possible cost must be determined for the optimal network selection for handover. The proposed approach could achieve load balancing in the target networks, and prevents unnecessary handovers. Also, [21] proposed another predictive RSS-based method that addresses the QoS parameters based on dynamic network conditions in HetNets.

Liu et al., [22] proposed a scheme to achieve improved performance in terms of low number of unnecessary handovers and Ping-Pong effect avoidance, based on the predicted RSS and mobile velocity; but this scheme needs to address some more performance assessment measures such as handover latency. According to the proposed scheme, if the mobile node is attached to a WLAN, and its velocity is higher than a predefined threshold velocity, a handover towards the UMTS network is initialized to prevent connection loss. The authors in [23] proposed a mobile agent-based scheme to prevent service interruptions during horizontal and vertical handovers in a heterogeneous environment. To predict a handover using this method, RSS of different networks is compared, and whenever the handover prediction is completed, it exploits its context to select an optimal network from the ones available based on user service requirements and preferences. P. Pawar [24] proposed a similar context based scheme that collects the contextual information from fixed networks and mobile nodes for providing end-to-end QoS.

The author in [25] described a cross layer predictive vertical handover mechanism that uses the MIH protocol to support QoS; this scheme can manage connectivity issues in heterogeneous wireless networks. The authors in [26] designed an estimation mechanism that is highly dependent on user-predicted traveling time in order to decrease the number of unnecessary handovers. However, this method has high handover latency, although it reduces unnecessary handovers and the probability of handover failures.

These works are very affective, but a vast majority of them are based on current system state only (i.e., they focus solely on current QoS of the networks and current mobile nodes’ conditions). Handover decisions need to consider the probabilistic outcomes of future system states as the result of the current decision. Hence, the integration of various intelligence aspects and prediction techniques is necessary in the decision function.

This study aims to propose a novel prediction algorithm that exploits the prediction model by using the imperialist competition algorithm (ICA), radial basis function (RBF) and firefly algorithm (FFA), to meet the above stated requirements. The purposes of the IRBF-FFA algorithm for prediction are threefold: (1) to serve as a validation algorithm for prediction; (2) to decrease the number of unnecessary handover, and prevent the ‘Ping-Pong’ effect; and (3) to improve the selection of the best candidate access point among various access technologies. ANNs are a good choice to address function prediction problems. This study presents a comparison based on two prediction methods, namely, the IRBF–FFA model; and support vector machines (SVM) and multilayer perceptron (MLP) models, to predict the RSSI. However, for all three cases, by proper training, weights and bias are determined to guarantee a specified performance goal.

**Fig 1:**
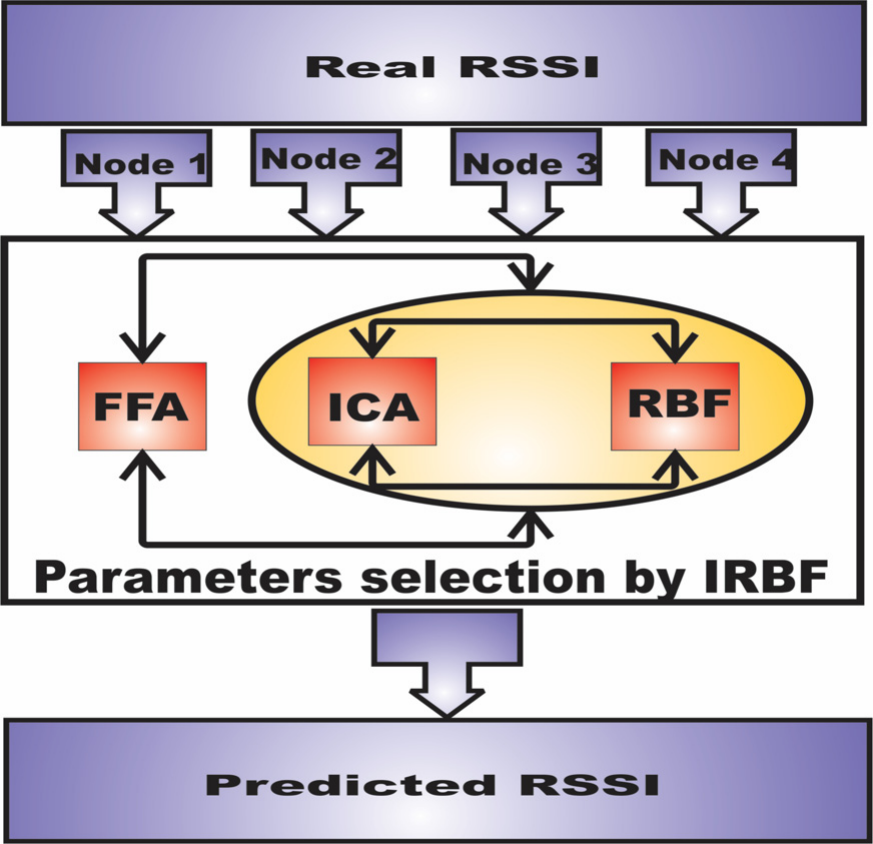
Diagram of IRBF-FFA.

More importantly, this comparison evaluates the aforementioned methods from different aspects, including root mean square (*RMSE*), coefficient of determination (*R*^2^) correlation coefficient (*r*) and means absolute percentage error (*MAPE*). The results suggest that IRBF–FFA has better performance; this is subsequently discussed. The proposed method is a step towards future computer-based optimization methods, where huge uncertainties by the optimization algorithm must be avoided. To do this, the imperialist competition algorithm (ICA) is used to train the radial basis function (RBF), combined with the Firefly Algorithm, to predict the optimal solution. Fig 1 presents a schematic diagram of the proposed IRBF–FFA model based on scanned RSSI as the considered input parameters.

The paper is outlined as follows. The materials and methods are described in Section 2. In Section 3, the proposed prediction method based on IRBF–FFA is explained. Section 4 introduces the different neural modelling methods for performance evaluation. Section 5 analyzes and discuses the performance of the algorithm through simulation results. Finally, section 6 concludes.

## Materials and Methods

A prediction technique for vertical handover using the IRBF–FFA algorithm is presented in this study. The RSSI is a common metric used in handover decision-making [27].

The initial RSSI is used to identify the existence of wireless networks. In heterogeneous wireless environments, when the mobile device senses more than one wireless network at the same time, the network selection with the best QoS plays an important role as the main problem. In the proposed model, the scanning phase first identifies the physical layer metric (RSSI) of available networks in heterogeneous environments to perform intelligent prediction.

**Fig 2:**
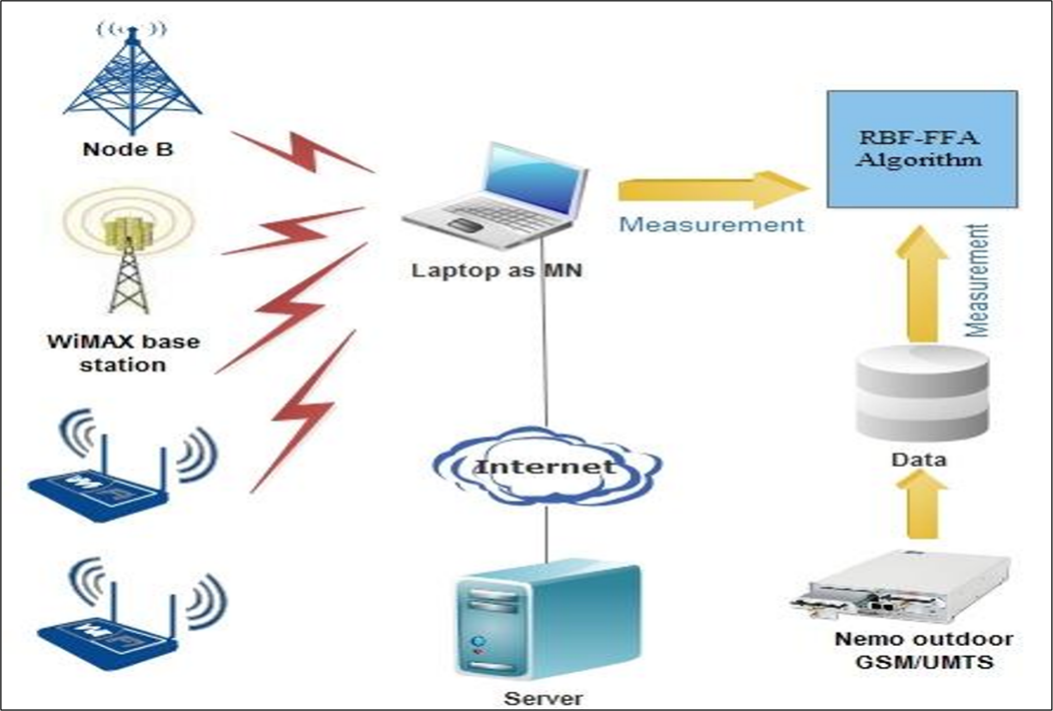
The architecture of the testbed.

For this purpose, we analyze the quality and distance for each AP in the range by scanning RSSI via mobile node (MN). When the MN changes its location to an AP, the level of RSSI for that AP is also changed. The real-tested measurement is used to realize the RSSI level through the MN movement. The scenario to perform the measurements comprises one computer as a MN within different nodes used to offer the UMTS and WLAN services. The computer collects MAC/PHY level KPIs, application level QoS KPIs, and location information. The location information is retrieved from a GPS device, as well as from the measurement tool QoSMeT for outdoors and indoors systems. The QoSMeT is able to monitor a large set of application level QoS KPIs over a point-to-point connection. The engineering mode terminal incorporates the Nemo Handy application, which provides the terminal with powerful radio monitoring capability. Nemo Handy provides extensive network parameters and exchanged signaling messages captured over data transfers. The logged measured data has been processed using the Nemo Outdoor software tool. The testbed enables both real-time offline measurements. A commercially available network-monitoring tool (Nemo Outdoor) was used to provide offline measurements for validation purposes. The server combines the incoming information with network planning information, and provides the prediction algorithm with the aggregated data. The architecture of the testbed is depicted in Fig 2.

The IRBF–FFA Model has been designed in this paper as a novel prediction model to construct a mathematical function that has the best fit to a series of data points for RSSI value in UMTS and WLAN networks. In the scanning phase, the received signal strength indicator parameter is measured as the main input to the IRBF–FFA Model to predict the received signal strength value for the four access points includes UMTS, WiMAX and two Wi-Fi in an intelligent approach. The RSSI has varying values during MN movement during a span of 15 Sec.

**Fig 3:**
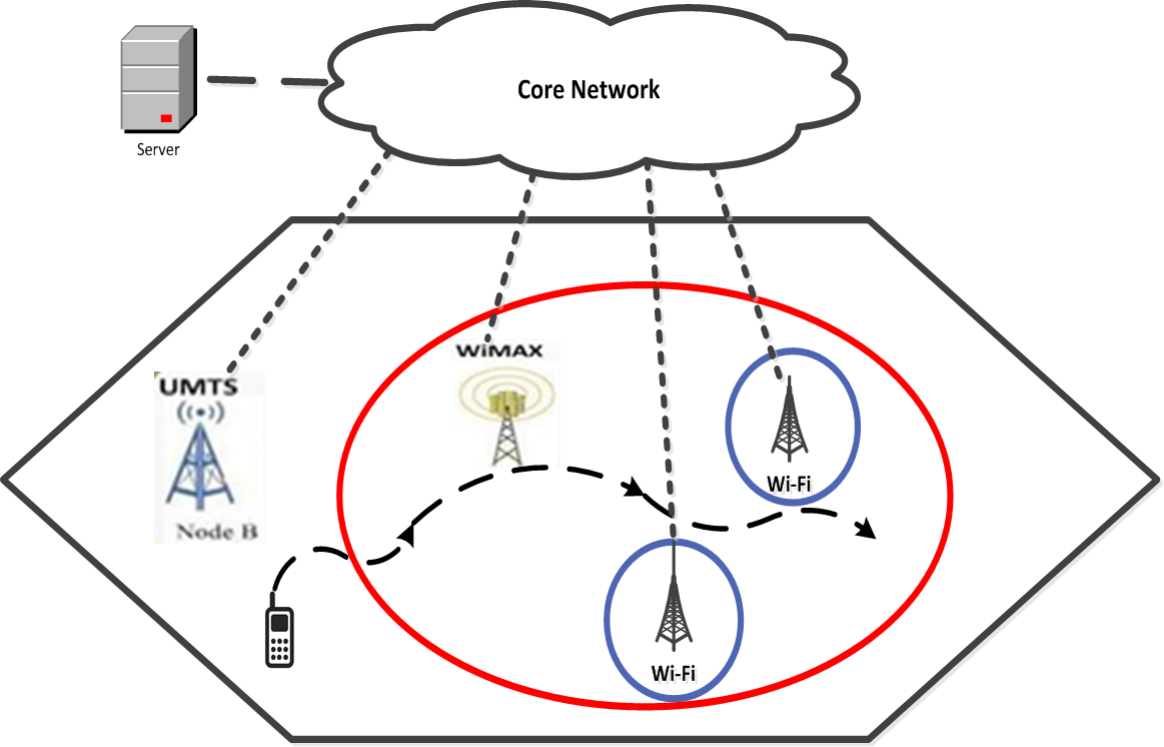
Simulation scenario.

The performance of the proposed algorithm has been assessed in a scenario in which the MN moves with a constant speed along a straight path from the area covered by UMTS to the one covered by WiMAX, and then roams to the area covered by Wi-Fi. Clearly, with the increase of distance, the average RSS, ABR, SNR and throughput are reduced, while BER is increased. The Simulation scenario is shown in Fig 3.

### Radial basis function (RBF)

**Fig 4:**
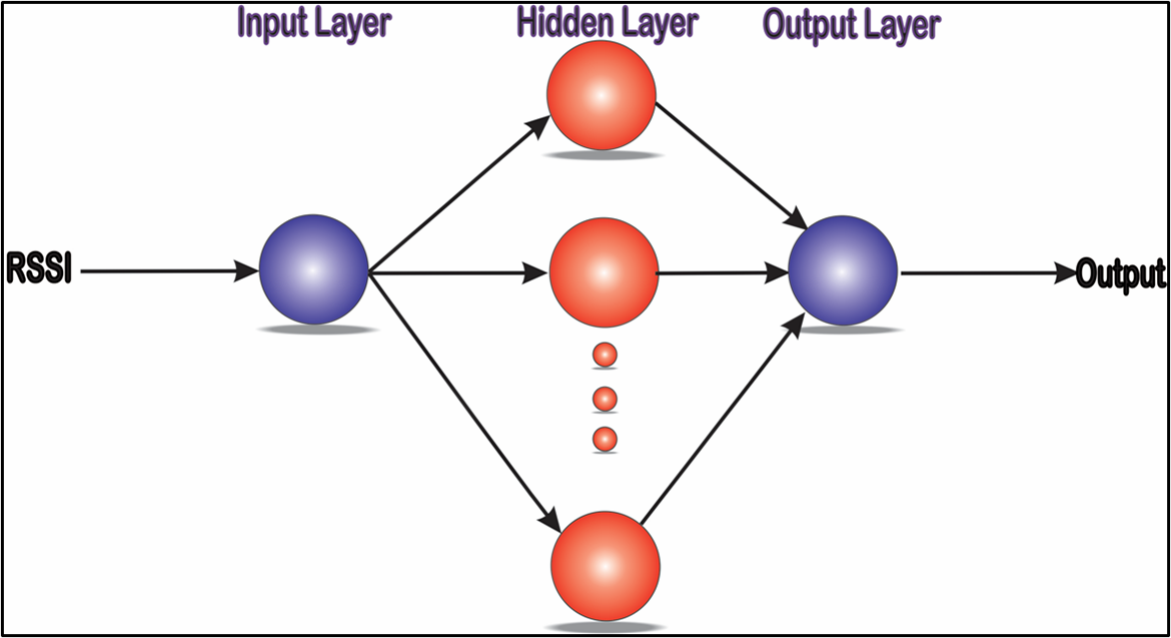
RBF Neural Network Structure.

Artificial Neural Networks (ANNs) have widely been used to develop, optimize, estimate, predict and monitor complicated systems. A new and effective feed forward neural network with three layers, called a radial basis function (RBF) neural network, has fine characteristics of approximation performance and the global optimum [28]. The RBF network generally consists of the input layer, the hidden layer and the output layer. Each neuron in the input layer is responsible for transferring the recorded signal to the hidden layer. In the hidden layer, we often use the radial basis function as the transfer function, while we usually adopt a simple linear function in the output layer. The RBF program was implemented in MATLAB. The RBF neural network with three layers is displayed in Fig 4. RBF was chosen because it has relatively good computational performance, simplicity, reliability and a high level of adaptation to optimization and other adaptive methods. Moreover, it is able to adapt and handle complicated parameters [29].

The RBF network basically comprises three layers (input layer, hidden layer and output layer). The ANN executes the nominal computation to offer an output. The computation comprises one-pass arithmetic steps. No iterative and nonlinear computations are complicated in offering an output. We have chosen RBF networks because it has a simple three layer design. The number of neurons in the hidden layer is set to 15; the MSE is set to 0.1 according to the actual training process; and the σ parameter (the width of the RBF) is set to 0.02.

The main advantage is that RBF has a hidden layer that comprises nodes called RBF units. Each RBF includes main factors that designate the location, deviation or width of the function’s center. The hidden component processes the distance from the input data vector and the center of its RBF. If the distance from the specific center to the input data vector is zero, then RBF has its own peak. However, if the distance increases, the peak of the RBF steadily declines.

In the RBF, hidden layers have different sets of weights that are divided into two sets. These weights can connect the hidden layer to the input layer, and the hidden layer to the output layer, as linkages. The subjects of the basis functions fixed into the weights those connect to the input layer. Since the hidden units are nonlinear, the outputs of the hidden layer can be merged linearly, and subsequently, processing becomes faster. The output of the network is computed using the following formula [30]:

$$y_{k}(x)=\sum_{j=1}^{N}w_{kj}\phi_{j}(x)+w_{k0}$$ (1)

where *N* is the number of basic functions, *w*_*kj*_ represents a weighted connection between the basis function and output layer, *x* is the input data vector, and *ϕ*_*j*_ is the nonlinear function of unit *j*, which is typically a Gaussian of the following form [30]:

$$\phi_{j}(x)=\text{exp}(-\frac{{\left\|x-\mu \right\|}^{2}}{2\sigma_{j}^{2}})$$ (2)

where *x* and *μ* are the input and the center of the RBF unit respectively, while *σ*_*j*_ is the spread of the Gaussian basis function [30]. The weights can be optimized by the LMS algorithm once the centers of RBF units are determined. The centers are selected either randomly, or by clustering algorithms.

### Imperialist Competitive Algorithm

The Imperialist Competition Algorithm (ICA) is a new global search algorithm based on the socio-political strategy for optimizing different tasks [31]. It is worthy to note that this algorithm has two great advantages in terms of convergence rate and good global search. ICA can be useful in different domains. For example, ICA is used to design an optimal controller [32]; to converse analysis of an ANN to describe the types of materials in the testing step [23]; or to find the Nash equilibrium point of various games [33]. In this study, ICA is used in interpolation of the RBF. In other words, we train the RBF by ICA as an optimization problem to forecast RSSI. ICA was implemented in this study to optimize the connection weights of the RBF system.

**Fig 5:**
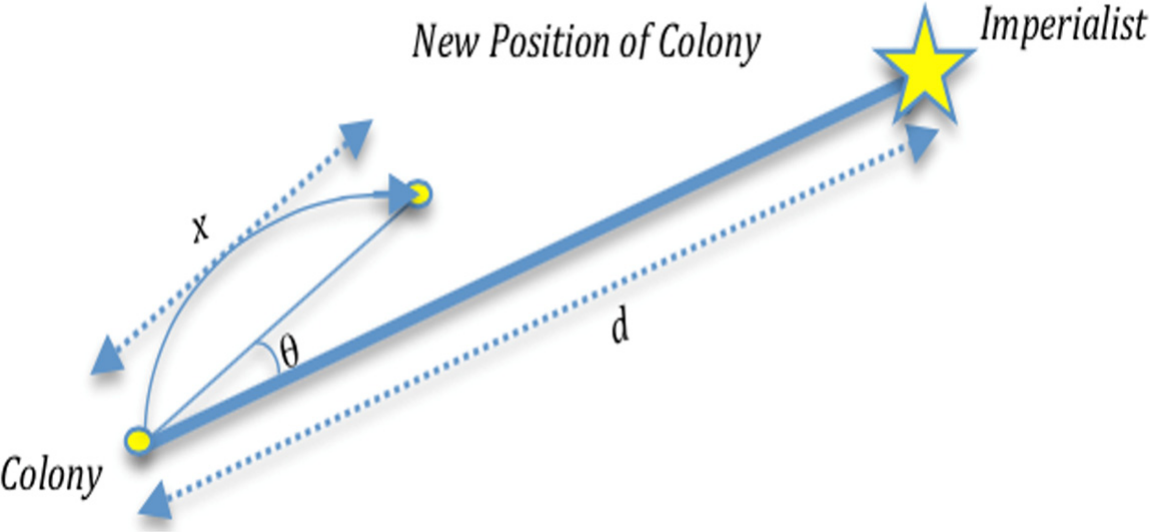
Movement of colony toward its relevant imperialist.

In the first step, ICA starts with a defined population. All individuals of the population are called countries. After initialization, the colonies start moving toward their relevant imperialist country. Fig 5 shows the movement of a colony towards the imperialist, where *θ* and *x* are random numbers with uniform distribution, and *d* is the distance between the colony and the imperialist.

**Fig 6:**
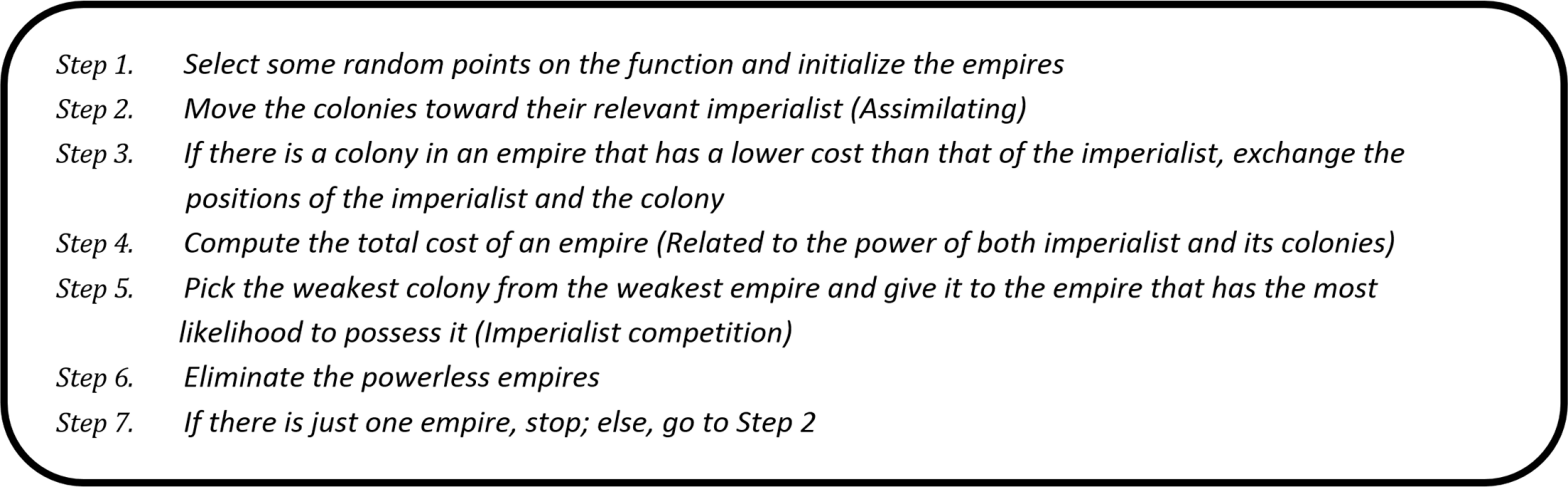
Pseudo code for the Imperialist Competition Algorithm (ICA).

The power of each country must be computed based on cost function, and then some of the countries are selected as the best with the lowest cost, called imperialists; the rest play the role as their colonies. In the next stage, the colonies have a competition to achieve the relevant imperialist’s position. The total power of the empire is related to both the power of its imperialist, and their colonies. The number of kernel RBFs was set to 10. Also, the MSE was used as cost function in the ICA. The Pseudo code for the ICA is shown in Fig 6.

### Firefly optimization algorithm

The Firefly Algorithm (FFA) is a meta-heuristic search algorithm based on the social dashing behavior of fireflies in nature [34,35]. In the FA, there are two important issues: the difference of light intensity, and the formulation of the attractiveness. We can consider that the attractiveness of a firefly is assessed by its light intensity that in turn is related with the encoded objective function. For simplicity, the light intensity L (d) varies with the distance d monotonically and exponentially, based on Eq (3).

$$L=L_{0}e^{-\gamma d}$$ (3)

where light intensity and absorption coefficient are presented by *L*_0_ and *γ* respectively. As a firefly’s attractiveness is proportional to the light intensity seen by adjacent fireflies, we can now define the attractiveness *β* of a firefly as follows:

$$\beta =\beta_{0}e^{-\gamma d^{2}}$$ (4)

where *β*_0_ is the attractiveness at *d* = 0. The distance between any two fireflies *i* and *j* at *X*_*i*_ and *X*_*j*_ can be the Cartesian distance *d*_*ij*_ = ‖*X*_*i*_−*X*_*j*_‖^2^ or the 2-norm. The movement of a firefly *i* attracted to another more attractive (brighter) firefly *j* is determined as follows:

$$X_{i}=X_{i}+\beta_{0}e^{-\gamma d^{2}}\;(X_{j}-X_{i})+\alpha \varepsilon_{i}$$ (5)

where the second term is due to the attraction, while the third term is randomization with the vector of random variables *ε*_*i*_ being drawn from a Gaussian distribution.

**Fig 7:**
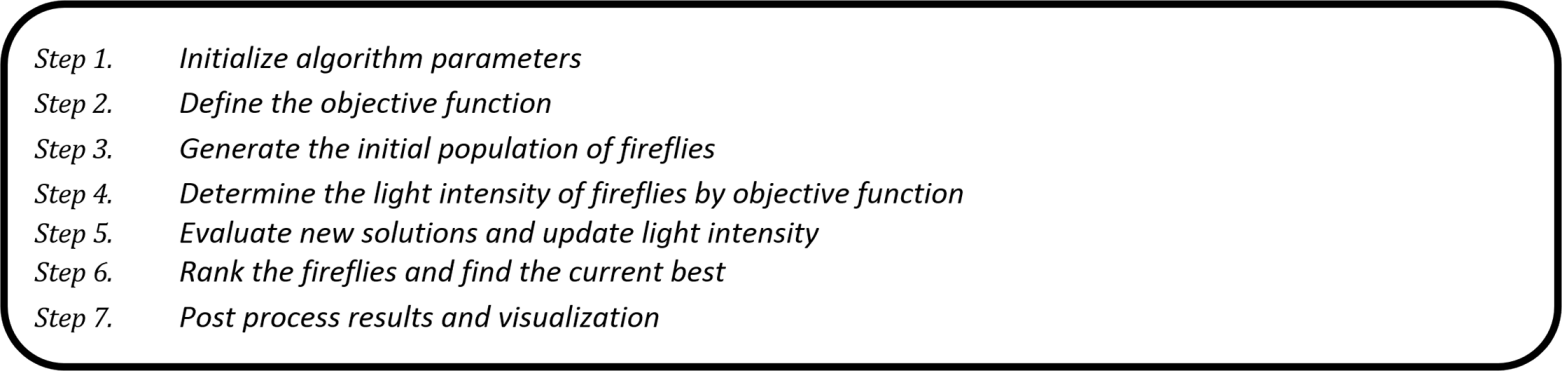
Description of the Firefly Algorithm (FFA).

In this study, we have developed a novel prediction algorithm for prediction of RSSI to boost handover decision making via hybridization of IRBF and the FFA. We used the FFA for determining optimal RBF solutions. To achieve this, four nodes were distributed in different locations to analyze the influence of distance on the capability of the developed method. The general description of the FFA is shown in Fig 7. The Matlab codes for implementing the ICA-RBF are shown in Appendix A.

## RBF Parameters Selection Using ICA and FFA

**Fig 8:**
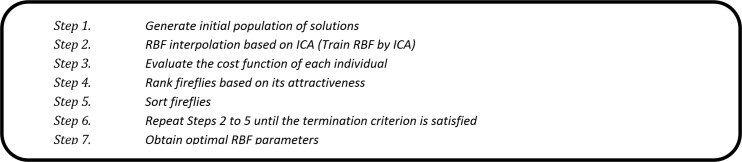
Pseudo code for the IRBF-FFA.

ANNs with radial basis function (RBF) based on ICA have been utilized to interpolation in order to approximate the solution, and are then combined with the FFA to estimate RSSI data. In this section, the explanation of the experiment for the IRBF–FFA model is shown. It should be mentioned that, here, the number of kernel RBFs was set to 10. Also, the MSE was used as a cost function in the ICA. The ability of the IRBF to make good predictions is related to input parameters selection. The RSSI data are inserted as inputs into IRBF in order to examine the best prediction using this method. The pseudo code of IRBF–FFA to determine the optimal RBF parameters is shown in Fig 8.

**Fig 9:**
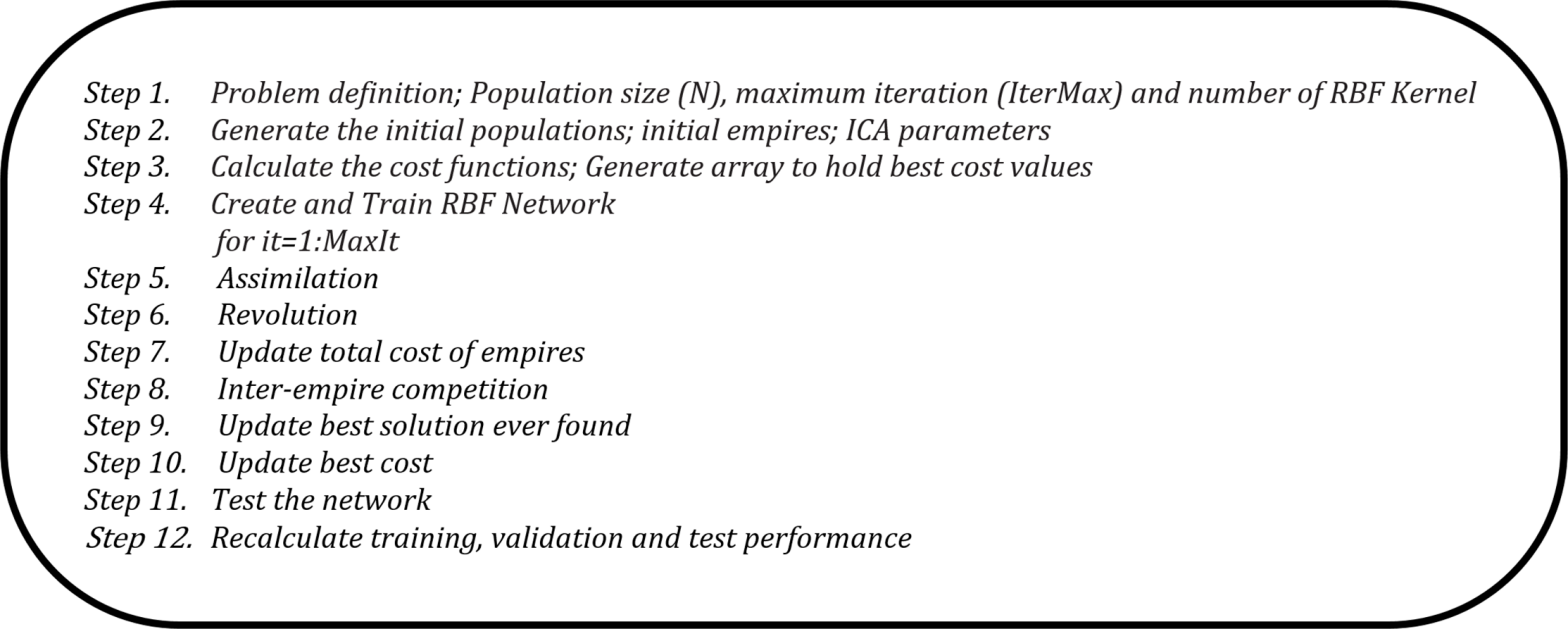
Hybrid of ICA and RBF.

The pseudo code of the hybrid ICA and RBF is shown in Fig 9. In this combination, we train the RBF using ICA. In other words, in order to improve the accuracy of the prediction, the responsibility of RBF’s training is considered. For the ICA-RBF algorithm, we have provided a brief introduction. Next, the operations of the main functions, namely, *CreateInitialEmpires*, *AssimilateColonies*, *DoRevolution*, *InterEmpireCompetition* and *IntraEmpireCompetition*, are explained in pseudocode format (Appendix A-E) to facilitate the implementation and use of such algorithms by researchers and practitioners.

## Model Performance Evaluation

Various neural modelling methods, namely, SVMs and MLPs are tested to model IRBF–FFA. To evaluate the performance of the proposed model and two famous prediction methods MLP and SVM; some statistical indicators were examined as root mean squared error (*RMSE*), coefficient of determination (*R*^2^), correlation coefficient (*r*) and mean absolute percentage error (*MAPE*). Structurally, the evaluated networks consist of a single input and output layer; and a single hidden layer for MLP, IRBF–FFA and SVM. We have postponed the evaluation and comparison of the approaches until Section 5.

### Support Vector Machine (SVM)

A SVMs model is a supervised learning method that is used for classification and regression analysis. In this study, in order to predict RSSI using SVMs, the RSSI of four nodes as inputs are mapped and generated in the first step of the training stage by kernel functions, and then the results of this stage are applied to compute the related weights. All of the aforementioned steps should be repeated in the testing stage; however, in the training step, the trained RSSI use only the testing RSSI for mapping. Afterwards, it will be applied on the kernel function. In other words, in the training stage, the inputs of the SVMs model are RSSI of four sources, which generate mapping vectors via kernel functions and outputs that are presented as derived weights and bias. During the testing stage, the inputs of the SVMs model are as follows: the RSSI of the testing stage, derived weights, bias and also the outputs are predicted RSSI.

### Multilayer Perceptron (MLP)

**Fig 10:**
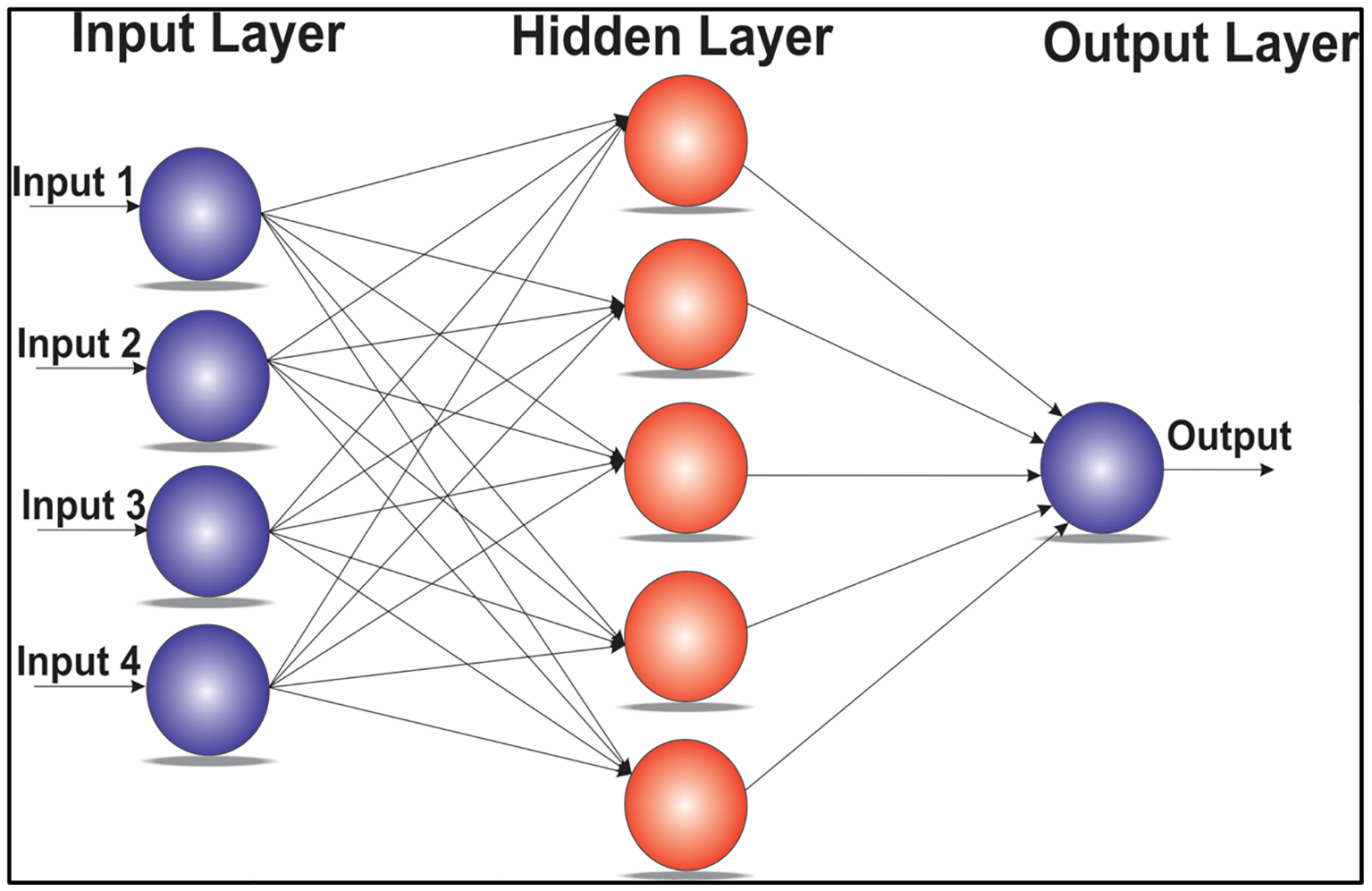
MLP network topology.

From past two decades, ANNs have become a popular research topic in various domains [36]. The number of neurons selected plays an important role in the overall process; because a high number of neurons can potentially lead to inadequate generalization due to the over fitting; while a low number of neurons can potentially lead to poor performance. Thus, the number of neurons should be selected suitably, based on a trial and error process. In this study, a MLP with a single hidden layer is used, as this is a general approximation when enough hidden neurons are employed. The MLP network topology with a single hidden layer is shown in Fig 10.

## Results and Discussions

This experiment is for the case of a vertical handover between a 3G network and WLANs. The scenario simulated in the MATLAB is composed of the UMTS, WiMAX and IEEE 802.11 APs. The WiMAX is based on the IEEE 802.16 standard. The capacity of a 3G Universal Mobile Telecommunications System is 384 kbps. All links except for the wireless links have a capacity of 100Mbps each. Ad hoc on-demand distance vector (AODV) protocol is used as the reactive routing protocol [37], [38]. This protocol offers quick convergence when the ad hoc network topology changes (typically, when a node moves in the network). We consider that the velocity from 1 to 25m/s, the number of UMTSs equals 1, arrival rate of Poisson distribution is 6 to 16, and the bandwidths of B3G/UMTS and WLAN are 384kb/s and 54 Mb/s, respectively. The topology covers an area of 2000 m in length and 2000m in width.

As shown in Fig 3, the mobile node can be at a fixed time in the coverage area of the UMTS. Nevertheless, due to movement, it can travel into the areas that cover more than one access network, that is, simultaneously within the coverage areas of, for example, a UMTS BS and WiMAX access point. Multiple WLAN coverage areas are usually comprised within a UMTS coverage area. Since the WLAN has a lower coverage range when the mobile user is moving out of a WLAN area, the existence of an accurate and timely handoff decision to maintain the connectivity before the loss of the WLAN access is necessary. The user could move into the regions covered by a UMTS network and then the user could move into a WiMAX area to achieve a higher QoS at the lowest cost. Therefore, the user changes the connection to the WiMAX access point. Then the mobile node roams to the area covered by Wi-Fi.

The mobile node associated with the UMTS or WLANs monitors and measures *D*_*RSS*_, which is the diversity of the received signal strength between the networks of the nearby WLANs/UMTS to check whether an access network with high data rate is offered. The proposed method using the IRBF–FFA prediction is compared to the SVMs and MLP. The simulation considers two classes of traffic, that is, constant bit rate (CBR) and variable bit rate (VBR). Some applications produce VBR (variable bit rate) traffic streams, while constant applications produce CBR traffic streams. The CBR traffic stream is easy to model and to predict its impact on the performance of the network. Data rates of CBR of B3G and WLAN are 50 and 200 (Kbps) and low and high-level data rates of VBR for B3G and WLAN are 10 (Kbps) and 1.6 (Mbps), respectively [19]. The route as demonstrated in the Fig 3 shows the trajectory of a moving UE which is assumed to follow a certain pattern, where the simulation parameters of the mobile user are given in the (Table 1).

**Table 1:** Simulation parameters and channel characteristics.

Parameter	WiMAX	UMTS	Wi-Fi
Cell Radius (m)	100	250	10
Transmit Power (Watts)	0.01	0.5	0.036
RSS_min_Th [dBm]	-95	-98	-95
MS speed [m/s]	10	10	10
Frequency band [GHz]	2.4	8e+8	2.4
Simulation duration [s]	100	100	100
Fading (Ϭ)	3	4	4

In this section, we attempt to demonstrate the importance of each independent input variable on the output. Some experimental works were executed to conduct the evaluation of proposed model. The root-mean-square error (*RMSE*), coefficient of determination(*R*^2^), correlation coefficient (*r*) and mean absolute percentage error (*MAPE*) served to evaluate the differences between the predicted and actual values for both SVMs models.

**Fig 11:**
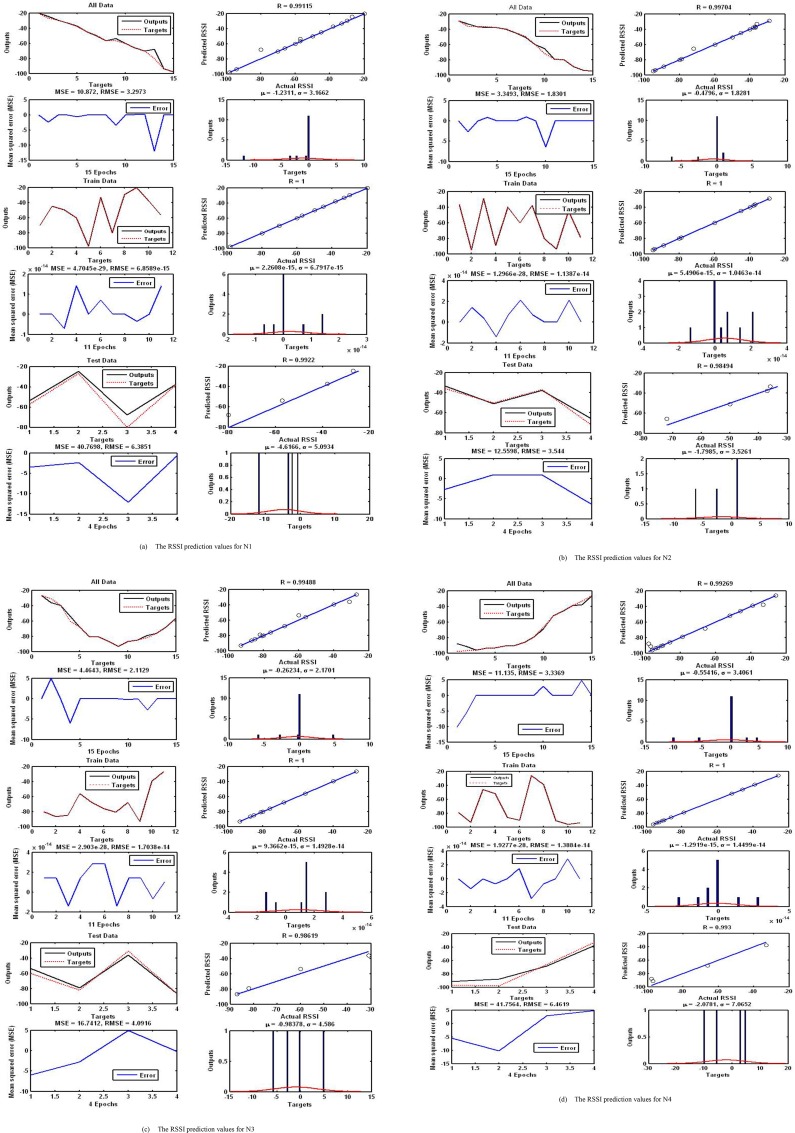
The plots of IRBF–FFA model predicted versus actual values for training, testing and all data sets for N1, N2, N3, and N4. (a) The RSSI prediction values for N1. (b) The RSSI prediction values for N2. (c) The RSSI prediction values for N3. (d) The RSSI prediction values for N4.

The radial basis ANN model was trained to minimize the MSE with RSSI parameter as input, and the desired output (predicted RSSI). To design and verify the reliability of the proposed model, the dataset was divided into two different sets: training (80%) and test (20%). There is no overlap with the test data during training. Afterwards, when the training process is done, the reliability and over fitting of the network were verified with the test data. The overall performance of the proposed models in estimating the RSSI of four nodes has been graphically depicted in Fig 11.

In order to acquire correct assessment, the RBF models are tested with a data set that has not been used during the training process. The real and predicted RSSI values for four Aps during 15 seconds have been stated in Table 2. According to (Table 2), the IRBF–FFA model can estimate the RSSI value quickly (about 800 ms before the actual time).

**Table 2:** Real and predicted RSSI values.

Time	Real RSSI				Predicted RSSI by IRBF–FFA			
	(UMTS) N1	(WiMAX) N2	(Wi-Fi) N3	(Wi-Fi) N4	(UMTS) N1	(WiMAX) N2	(Wi-Fi) N3	(Wi-Fi) N4
1	-20	-29	-27	-98				
1.7					-25.8	-35.1	-30.6	-96.4
2	-27	-36	-31	-97				
2.5					-29.6	-36.8	-38.4	-95.3
3	-30	-37	-40	-96				
3.2					-32.8	-36.9	-55.2	-93.6
4	-33	-37	-60	-94				
4.4					-38.1	-37.5	-66.1	-94.8
5	-38	-38	-68	-93				
5.6					-44.3	-40.4	-79.9	-92.5
6	-45	-40	-80	-91				
6.8					-48.9	-45.1	-80.2	-89.9
7	-50	-45	-81	-90				
7.5					-55.6	-49.9	-86.5	-86.6
8	-57	-50	-87	-86				
8.6					-56.9	-58.2	-92.5	-79.3
9	-57	-60	-93	-79				
9.2					-59.5	-70.8	-86.2	-65.4
10	-60	-72	-87	-66				
10.4					-65.3	-78.8	-85.3	-53
11	-66	-79	-85	-52				
11.6					-70.3	-80.1	-82.7	-46.6
12	-70	-80	-82	-46				
12.8					-79.9	-89.3	-75.4	-39
13	-80	-89	-76	-39				
13.4					-90.9	-93.6	-69.6	-34.1
14	-94	-94	-68	-33				
14.6					-97.6	-94.8	-57.1	-26.1
15	-98	-95	-56	-26				

In order to assess the performance of fit in our RBF–FFA (Fig 5), residual analysis has been modified and used. This is to justify in the manner in which the IRBF–FFA is able to predict new RSSI values, with a great degree of certainty, resulting from extremely variable RSSI data collected from APs. To evaluate the performance of the RBF–FFA, three statistical estimators were used, which are as follows: the mean squared error (*MSE*) shown in Eq (6), the coefficient of determination (*R*^2^) shown in Eq (7) and the root mean square error (*RMSE*) shown in Eq (8). Note that if the *RMSE* is zero, the method has outstanding performance.

$$MSE=\frac{1}{r}\sum_{i=1}^{r}{\left(V_{pi}-V_{ai}\right)}^{2}$$ (6)

$$R^{2}=1-\frac{\sum_{i=1}^{r}{\left(V_{pi}-V_{ai}\right)}^{2}}{\sum_{i=1}^{r}{\left(V_{pi}-V_{av}\right)}^{2}}$$ (7)

$$RMSE=\sqrt{\frac{1}{r}\sum_{i=1}^{r}{\left(V_{pi}-V_{ai}\right)}^{2}}$$ (8)

where *r* is the number of points; *V*_*pi*_ is the estimated value; *V*_*ai*_ is the actual value; and *V*_*av*_ is the average of the actual values. The coefficient of determination, *R*^2^ of the linear regression line between the estimated values of the neural network model, and the required output, was also used as a measure of performance. The closer the *R*^2^ value is to 1, the better the model fits the actual data [39]. This measurement determines the degree of success the fit is in describing the change of the data. Expressed differently, *R*^2^ is the square of the correlation between the response values and the predicted response values. It is also referred to as the square of the multiple correlation coefficients, and the coefficient of multiple determinations. Fig 6 shows more details for N1, N2, N3 and N4. It is worthy to note that, the suitable selection of initial weights may cause the local minimum data. In order to prevent this unfavorable phenomenon, 30 runs for each method were applied. In each run, different random values of initial weights were measured. Finally, in RBF, the best-trained network that had a minimum *MSE* of validation data was selected as the trained network. The estimation performance of IRBF–FFA, SVM and MLP were assessed by *R*^2^ and *MSE*. The output values are stated in Table 3. Table 3 shows a comparison of IRBF–FFA with SVMs and MLP. This table shows the results of 30 independent runs with Iteration = 100.

**Table 3:** The performances of IRBF–FFA model based on R2 and RMSE compares to other methodologies.

Run no	IRBF–FFA								SVM								MLP							
	R^2^				RMSE				R^2^				RMSE				R^2^				RMSE			
	N1	N2	N3	N4	N1	N2	N3	N4	N1	N2	N3	N4	N1	N2	N3	N4	N1	N2	N3	N4	N1	N2	N3	N4
1	0.99	0.98	0.99	0.99	0.69	0.78	0.69	0.63	0.79	0.88	0.89	0.79	2.69	1.78	1.69	1.63	0.93	0.72	0.78	0.72	1.36	1.98	1.98	1.98
2	0.98	0.98	0.98	0.99	0.78	0.78	0.78	0.69	0.88	0.88	0.88	0.79	1.78	1.78	1.78	1.69	0.91	0.72	0.66	0.82	0.98	1.98	1.56	1.90
3	0.97	0.98	0.99	0.99	0.79	0.59	0.69	0.69	0.77	0.88	0.89	0.79	1.79	1.59	1.69	1.69	0.80	0.69	0.67	0.72	0.90	1.02	0.98	1.02
4	0.99	0.99	0.99	0.99	0.69	0.69	0.69	0.69	0.89	0.89	0.89	0.79	1.69	1.69	1.69	1.69	0.92	0.72	0.68	0.80	0.98	1.98	1.43	1.02
5	0.99	0.98	0.98	0.99	0.69	0.79	0.59	0.59	0.79	0.88	0.88	0.79	1.69	2.79	1.59	2.59	0.88	0.70	0.78	0.86	0.99	1.36	1.98	1.98
6	0.97	0.98	0.99	0.99	0.79	0.69	0.69	0.69	0.77	0.88	0.89	0.79	1.79	2.69	1.69	1.69	0.86	0.70	0.64	0.82	1.02	0.98	1.99	1.98
7	0.99	0.98	0.99	0.99	0.69	0.69	0.69	0.69	0.79	0.88	0.89	0.79	2.69	1.69	1.69	1.69	0.93	0.68	0.77	0.82	1.36	1.36	1.36	0.98
8	0.99	0.98	0.98	0.99	0.69	0.59	0.59	0.69	0.89	0.88	0.88	0.79	2.69	2.59	2.59	1.69	0.93	0.72	0.68	0.74	1.36	1.98	0.98	0.99
9	0.97	0.99	0.99	0.98	0.79	0.59	0.69	0.89	0.87	0.89	0.89	0.78	2.79	1.59	2.69	1.89	0.91	0.72	0.76	0.74	0.98	1.98	1.43	1.02
10	0.99	0.98	0.99	0.99	0.69	0.69	0.69	0.69	0.89	0.88	0.89	0.79	2.69	169	1.69	1.69	0.90	0.69	0.67	0.72	1.43	1.33	1.36	1.36
11	0.99	0.98	0.99	0.99	0.69	0.69	0.69	0.69	0.89	0.88	0.89	0.99	2.69	1.69	1.69	1.69	0.72	0.72	0.68	0.70	1.35	1.98	1.36	1.98
12	0.99	0.98	0.99	0.99	0.69	0.69	0.69	0.69	0.89	0.88	0.89	0.89	1.69	1.69	1.69	2.69	0.98	0.70	0.68	0.76	0.99	1.33	1.89	1.98
13	0.99	0.99	0.99	0.98	0.59	0.69	0.59	0.80	0.79	0.89	0.89	0.88	1.59	2.69	1.59	2.80	0.96	0.70	0.64	0.72	1.33	1.36	0.98	1.89
14	0.99	0.99	0.99	0.99	0.69	0.69	0.69	0.59	0.79	0.89	0.89	0.79	1.69	2.69	2.69	2.59	0.93	0.68	0.67	0.72	1.36	0.98	1.36	1.36
15	0.99	0.98	0.99	0.98	0.69	0.69	0.69	0.79	0.79	0.88	0.89	0.78	1.69	2.69	2.69	2.79	0.93	0.72	0.58	0.72	1.36	1.98	0.98	1.36
16	0.99	0.98	0.99	0.99	0.58	0.69	0.79	0.69	0.89	0.88	0.89	0.99	1.58	1.69	1.79	2.69	0.93	0.72	0.56	0.83	1.36	1.98	0.99	1.89
17	0.99	0.98	0.99	0.99	0.69	0.59	0.69	0.69	0.89	0.88	0.89	0.79	2.69	1.59	1.69	1.69	0.90	0.69	0.85	0.82	1.89	1.89	1.02	0.98
18	0.98	0.99	0.99	0.97	0.78	0.69	0.69	0.79	0.78	0.89	0.89	0.77	278	1.69	1.69	1.79	0.82	0.72	0.58	0.80	0.98	1.98	1.36	1.89
19	0.99	0.99	0.97	0.99	0.69	0.69	0.69	0.69	0.89	0.89	0.87	0.89	2.69	1.69	1.69	1.69	0.88	0.70	0.58	0.86	0.99	1.36	1.36	0.98
20	0.96	0.98	0.99	0.99	0.80	0.69	0.79	0.59	0.76	0.88	0.89	0.89	2.80	169	1.79	1.59	0.86	0.70	0.54	0.87	1.02	1.36	1.98	1.36
21	0.99	0.98	0.99	0.99	0.69	0.69	0.69	0.69	0.89	0.88	0.89	0.89	2.69	1.69	1.69	2.69	0.93	0.68	0.57	0.82	1.36	0.98	1.98	0.98
22	0.98	0.99	0.99	0.99	0.69	0.69	0.59	0.69	0.88	0.89	0.89	0.89	2.69	1.69	2.59	2.69	0.93	0.72	0.58	0.72	1.36	1.98	1.89	1.98
23	0.99	0.99	0.99	0.98	0.69	0.69	0.69	0.79	0.89	0.89	0.89	0.88	2.69	1.69	2.69	2.79	0.91	0.73	0.56	0.87	0.98	0.98	0.98	1.98
24	0.98	0.99	0.99	0.99	0.78	0.59	0.69	0.69	0.88	0.89	0.89	0.79	1.78	1.59	2.69	1.69	0.90	0.79	0.57	0.82	1.89	1.43	1.36	1.89
25	0.97	0.98	0.99	0.99	0.79	0.69	0.69	0.79	0.87	0.88	0.89	0.79	1.79	2.69	1.69	2.79	0.91	0.72	0.58	0.80	0.98	1.98	0.98	1.89
26	0.90	0.98	0.99	0.98	0.88	0.69	0.69	0.79	0.70	0.88	0.89	0.78	1.88	2.69	0.99	2.79	0.93	0.70	0.58	0.87	1.36	0.98	1.35	0.98
27	0.99	0.99	0.97	0.99	0.69	0.79	0.79	0.69	0.89	0.89	0.87	0.79	1.69	2.79	0.99	1.69	0.92	0.70	0.54	0.88	0.98	1.36	0.98	1.36
28	0.98	0.99	0.99	0.99	0.69	0.69	0.69	0.59	0.78	0.89	0.89	0.79	1.69	2.69	0.99	1.59	0.97	0.70	0.47	0.88	1.35	0.98	0.99	0.98
29	0.99	0.99	0.99	0.98	0.69	0.69	0.69	0.79	0.89	0.89	0.89	0.78	2.69	2.69	1.69	1.79	0.95	0.72	0.58	0.82	1.01	1.98	1.02	1.35
30	0.98	0.98	0.99	0.99	0.69	0.69	0.69	0.69	0.88	0.88	0.89	0.89	1.69	2.69	1.69	1.69	0.91	0.72	0.56	0.89	0.98	1.98	1.36	1.89

Table 3 shows *R*^2^ values of all data sets for the IRBF–FFA, SVM and MLP. It is clear that the fit is rationally suitable for all data sets with R-values of 1 for the IRBF–FFA. The SVM and MLP were found to be sufficient for estimation of the RSSI, whereas the IRBF–FFA model showed a significantly high degree of accuracy in the estimation of *R*^2^ between 0.97 and 0.99. Also, the root of *MSE* demonstrated that, the smaller the *RMSE* of the test data set, the higher the predictive quality. The assessment of the aforementioned models shows the suitable predictive capabilities of the IRBF–FFA model. This prediction method using IRBF–FFA helps to expect the RSSI value for each AP up to 800 ms distant from the actual value.

We also attempt to demonstrate the results of comparison based on the correlation coefficient (*r*) [40], [41] and mean absolute percentage error (MAPE) [42], which served to evaluate the differences between the predicted and actual values for the IRBF–FFA, SVM and MLP models. Table 4 shows the results of comparison based on (*r*) and (*MAPE*).

$$r=\frac{\sum_{i=1}^{n}\left(V_{pi}-\bar{V_{pi}}).(V_{ai}-\bar{V_{ai}}\right)}{\sqrt{\sum_{i=1}^{n}(V_{pi}-\bar{V_{pi}}).\sum_{i=1}^{n}\left(V_{ai}-\bar{V_{ai}}\right)}}$$ (9)

$$MAPE=\frac{1}{r}\sum_{i=1}^{n}\left|\frac{V_{pi}-V_{ai}}{V_{ai}}\right|\times 100$$ (10)

where *n* is the number of points; *V*_*pi*_ is the estimated value; *V*_*ai*_ is the actual value; and $\bar{V_{pi}}$ and $\bar{V_{ai}}$ are the mean value of *V*_*pi*_ and *V*_*ai*,_ respectively. The smaller value of *MAPE* has a better performance model, and vice versa, in the case of *r*.

**Table 4:** The performances of IRBF–FFA model based on r and MAPE compares to other methodologies.

Run no	IRBF–FFA								SVM								MLP							
	*r*				MAPE				*r*				MAPE				*r*				MAPE			
	N1	N2	N3	N4	N1	N2	N3	N4	N1	N2	N3	N4	N1	N2	N3	N4	N1	N2	N3	N4	N1	N2	N3	N4
1	0.99	0.98	0.99	0.99	0.26	0.29	0.22	0.26	0.89	0.78	0.99	0.89	0.69	0.78	0.69	0.63	0.93	0.72	0.78	0.72	1.36	1.98	1.98	1.98
2	0.98	0.98	0.98	0.99	0.28	0.28	0.22	0.27	0.88	0.88	0.88	0.89	0.78	0.78	0.78	0.69	0.91	0.72	0.66	0.82	0.98	1.98	1.56	1.90
3	0.97	0.98	0.99	0.99	0.26	0.28	0.24	0.27	0.87	0.78	0.89	0.89	0.79	0.59	0.69	0.69	0.80	0.69	0.67	0.72	0.90	1.02	0.98	1.02
4	0.99	0.99	0.99	0.99	0.27	0.29	0.22	0.26	0.89	0.89	0.89	0.89	0.69	0.69	0.69	0.69	0.92	0.72	0.68	0.80	0.98	1.98	1.43	1.02
5	0.99	0.98	0.98	0.99	0.26	0.30	0.25	0.27	0.88	0.78	0.88	0.89	0.69	0.79	0.59	0.59	0.88	0.70	0.78	0.86	0.99	1.36	1.98	1.98
6	0.97	0.98	0.99	0.99	0.26	0.30	0.25	0.26	0.87	0.88	0.99	0.99	0.79	0.69	0.69	0.69	0.86	0.70	0.64	0.82	1.02	0.98	1.99	1.98
7	0.99	0.98	0.99	0.99	0.29	0.30	0.22	0.28	0.89	0.78	0.89	0.89	0.69	0.69	0.69	0.69	0.93	0.68	0.77	0.82	1.36	1.36	1.36	0.98
8	0.99	0.98	0.98	0.99	0.28	0.29	0.24	0.26	0.89	0.88	0.88	0.89	0.69	0.59	0.59	0.69	0.93	0.72	0.68	0.74	1.36	1.98	0.98	0.99
9	0.97	0.99	0.99	0.98	0.26	0.28	0.22	0.26	0.87	0.89	0.89	0.88	0.79	0.59	0.69	0.89	0.91	0.72	0.76	0.74	0.98	1.98	1.43	1.02
10	0.99	0.98	0.99	0.99	0.26	0.29	0.24	0.28	0.89	0.88	0.89	0.98	0.69	0.69	0.69	0.69	0.90	0.69	0.67	0.72	1.43	1.33	1.36	1.36
11	0.99	0.98	0.99	0.99	0.28	0.28	0.24	0.27	0.89	0.88	0.89	0.89	0.69	0.69	0.69	0.69	0.72	0.72	0.68	0.70	1.35	1.98	1.36	1.98
12	0.99	0.98	0.99	0.99	0.26	0.28	0.22	0.26	0.89	0.88	0.89	0.89	0.69	0.69	0.69	0.69	0.98	0.70	0.68	0.76	0.99	1.33	1.89	1.98
13	0.99	0.99	0.99	0.98	0.28	0.28	0.24	0.26	0.89	0.89	0.89	0.88	0.59	0.69	0.59	0.80	0.96	0.70	0.64	0.72	1.33	1.36	0.98	1.89
14	0.99	0.99	0.99	0.99	0.26	0.29	0.24	0.27	0.89	0.89	0.89	0.89	0.69	0.69	0.69	0.59	0.93	0.68	0.67	0.72	1.36	0.98	1.36	1.36
15	0.99	0.98	0.99	0.98	0.26	0.29	0.23	0.26	0.89	0.88	0.89	0.88	0.69	0.69	0.69	0.79	0.93	0.72	0.58	0.72	1.36	1.98	0.98	1.36
16	0.99	0.98	0.99	0.99	0.29	0.30	0.22	0.28	0.89	0.88	0.89	0.89	0.58	0.69	0.79	0.69	0.93	0.72	0.56	0.83	1.36	1.98	0.99	1.89
17	0.99	0.98	0.99	0.99	0.26	0.30	0.24	0.26	0.89	0.88	0.89	0.89	0.69	0.59	0.69	0.69	0.90	0.69	0.85	0.82	1.89	1.89	1.02	0.98
18	0.98	0.99	0.99	0.97	0.26	0.28	0.22	0.26	0.88	0.89	0.89	0.87	0.78	0.69	0.69	0.79	0.82	0.72	0.58	0.80	0.98	1.98	1.36	1.89
19	0.99	0.99	0.97	0.99	0.28	0.29	0.21	0.27	0.89	0.89	0.87	0.89	0.69	0.69	0.69	0.69	0.88	0.70	0.58	0.86	0.99	1.36	1.36	0.98
20	0.96	0.98	0.99	0.99	0.26	0.29	0.24	0.28	0.86	0.88	0.89	0.89	0.80	0.69	0.79	0.59	0.86	0.70	0.54	0.87	1.02	1.36	1.98	1.36
21	0.99	0.98	0.99	0.99	0.26	0.29	0.22	0.26	0.89	0.88	0.89	0.89	0.69	0.69	0.69	0.69	0.93	0.68	0.57	0.82	1.36	0.98	1.98	0.98
22	0.98	0.99	0.99	0.99	0.26	0.30	0.21	0.26	0.88	0.89	0.89	0.89	0.69	0.69	0.59	0.69	0.93	0.72	0.58	0.72	1.36	1.98	1.89	1.98
23	0.99	0.99	0.99	0.98	0.28	0.30	0.22	0.26	0.89	0.89	0.99	0.88	0.69	0.69	0.69	0.79	0.91	0.73	0.56	0.87	0.98	0.98	0.98	1.98
24	0.98	0.99	0.99	0.99	0.28	0.29	0.21	0.28	0.88	0.89	0.89	0.89	0.78	0.59	0.69	0.69	0.90	0.79	0.57	0.82	1.89	1.43	1.36	1.89
25	0.97	0.98	0.99	0.99	0.26	0.29	0.21	0.27	0.87	0.88	0.89	0.89	0.79	0.69	0.69	0.79	0.91	0.72	0.58	0.80	0.98	1.98	0.98	1.89
26	0.90	0.98	0.99	0.98	0.26	0.30	0.21	0.27	0.80	0.88	0.89	0.88	0.88	0.69	0.69	0.79	0.93	0.70	0.58	0.87	1.36	0.98	1.35	0.98
27	0.99	0.99	0.97	0.99	0.27	0.30	0.22	0.28	0.89	0.89	0.87	0.89	0.69	0.79	0.79	0.69	0.92	0.70	0.54	0.88	0.98	1.36	0.98	1.36
28	0.98	0.99	0.99	0.99	0.26	0.29	0.22	0.28	0.88	0.89	0.89	0.89	0.69	0.69	0.69	0.59	0.97	0.70	0.47	0.88	1.35	0.98	0.99	0.98
29	0.99	0.99	0.99	0.98	0.27	0.29	0.22	0.26	0.89	0.89	0.89	0.88	0.69	0.69	0.69	0.79	0.95	0.72	0.58	0.82	1.01	1.98	1.02	1.35
30	0.98	0.98	0.99	0.99	0.27	0.30	0.21	0.26	0.88	0.88	0.89	0.89	0.69	0.69	0.69	0.69	0.91	0.72	0.56	0.89	0.98	1.98	1.36	1.89

Tables 3 and 4 indicate that the IRBF–FFA model has the best capability in estimating the RSSI. Based on the results of comparison, it can be observed that the performance of the proposed model is different between the two considered approaches. After a comparison between the proposed IRBF–FFA model, and SVMs and MLP, the achieved demonstrate the former method is superior to the latter baseline.

**Fig 12:**
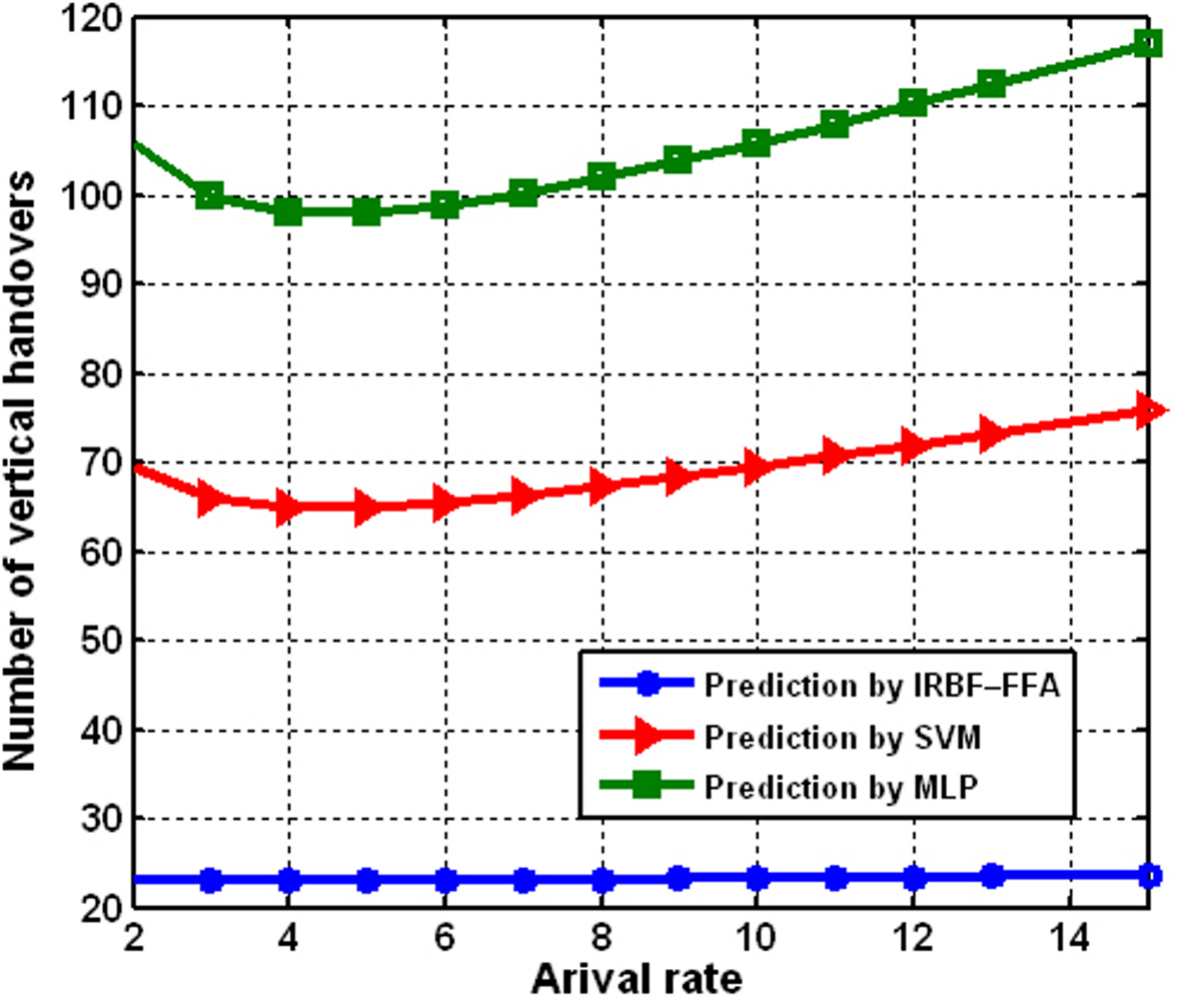
Comparing the number of vertical handoffs under various arrival rates.

Fig 12 shows the evaluation of the proposed approach by comparing the number of vertical handoffs. Clearly, the proposed prediction approach considerably improves the number of vertical handoffs. The 95% confidence intervals of the simulation results in Fig 12 are created from 30 independent runs. The number of vertical handoffs of all the approaches increases when the arrival rate increases. The proposed prediction model, the IRBF–FFA approach, results in the fewest vertical handoffs. Consequently, the IRBF–FFA approach outperforms other approaches in the number of vertical handoffs in heterogeneous wireless networks.

**Fig 13:**
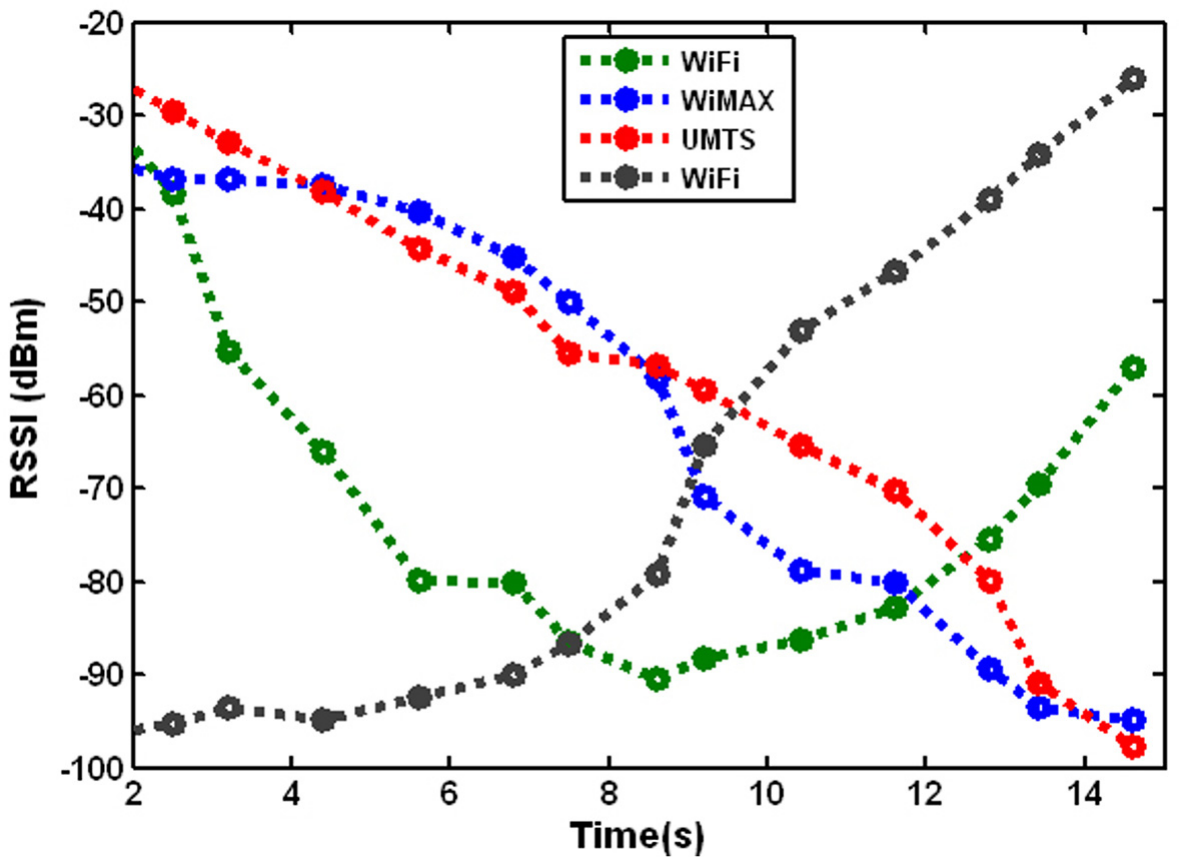
Predicted RSSI of MT in three different networks (WiMAX & Wi-Fi& UMTS).

The vertical handover process is based on the predicted RSSI value, which could set a smaller threshold whenever the predicted value is smaller than the threshold of the link layer triggering which might lead to being activated. So handover is performed before RSSI becomes weak to avoid ping-pong. Fig 13 displays the locations where handovers occur are shown in. It illustrates the proposed algorithm can execute accurate handovers and eliminate the ping-pong effect.

## Conclusion

In this study, a novel hybrid prediction model was proposed in order to predict the next RSSI value for APs that are in scanning area range. For this purpose, we integrated the imperialist competition Algorithm (ICA) to train the Radial basis function (RBF), combined with the Firefly Algorithm (FFA), to improve the prediction accuracy. The simulation studies measured RSSI data obtained from four different nodes. The main idea of the study focuses on the examination of the feasibility of the proposed hybrid technique in comparison to other techniques. To validate the precision of the developed IRBF–FFA model, its performance was compared to support vector machines (SVMs) and multilayer perceptron (MLP) models. After the analyses, the proposed model demonstrated better performance. The proposed method using IRBF–FFA can expect the RSSI value for every AP up to 800 ms distant from the actual value. So, MN is able to perform the handover procedure in a predictive mode within low latency. Our novel section scheme was aimed at making mobile nodes smart enough to be able to autonomously decide the best network using data prediction. The statistical indicator used for performance evaluation of the proposed model indicates lower values of RMSE and MAPE, and higher values of R^2^ and r, when compared to SVM and MLP models, for all the nodes considered. The IRBF–FFA model showed a significantly high degree of accuracy in the estimation of R^2^ between 0.97 and 0.99. The achieved results revealed that the proposed hybrid IRBF–FFA approach would be an appealing option to predict RSSI, since the results were favorable for all 30 runs, despite different node characteristics. Also, we compared the number of vertical handoffs under various arrival rates then we found that prediction by IRBF–FFA approach has better performance. The algorithm adopts predictive RSSI, capable save time that provides good ground to minimize ping-pong. Based on this, the proposed IRBF–FFA model can thus be allocated as an efficient approach for the accurate prediction of RSSI data.

## Appendix A: CreateInitialEmpires ()

function emp = CreateInitialEmpires()

    global ProblemSettings;

    global ICASettings;

    CostFunction = ProblemSettings.CostFunction;

    nVar = ProblemSettings.nVar;

    VarSize = ProblemSettings.VarSize;

    VarMin = ProblemSettings.VarMin;

    VarMax = ProblemSettings.VarMax;

    nPop = ICASettings.nPop;

    nEmp = ICASettings.nEmp;

    nCol = nPop-nEmp;

    alpha = ICASettings.alpha;

    empty_country.Position = [];

    empty_country.Cost = [];

    empty_country.Sol = [];

    country = repmat(empty_country,nPop,1);

    for i = 1:nPop

country(i).Position.m = unifrnd(VarMin.m,VarMax.m,VarSize.m); country(i).Position.sigma = unifrnd(VarMin.sigma,VarMax.sigma,VarSize.sigma);

country(i).Position.w = unifrnd(VarMin.w,VarMax.w,VarSize.w);[country(i).Cost country(i).Sol] = CostFunction(country(i).Position);

    end

    costs = [country.Cost];

    [~, SortOrder] = sort(costs);

    country = country(SortOrder);

    imp = country(1:nEmp);

    col = country(nEmp+1:end);

    empty_empire.Imp = [];

    empty_empire.Col = repmat(empty_country,0,1);

    empty_empire.nCol = 0;

    empty_empire.TotalCost = [];

    emp = repmat(empty_empire,nEmp,1);

    % Assign Imperialists

    for k = 1:nEmp

    emp(k).Imp = imp(k);

    end

    % Assign Colonies

    P = exp(-alpha*[imp.Cost]/max([imp.Cost]));

    P = P/sum(P);

    for j = 1:nCol

    k = RouletteWheelSelection(P);

    emp(k).Col = [emp(k).Col

    col(j)];

    emp(k).nCol = emp(k).nCol+1;

    end

    emp = UpdateTotalCost(emp);

    end

## Appendix B: AssimilateColonies(emp)

function emp = AssimilateColonies(emp)

global ProblemSettings;

CostFunction = ProblemSettings.CostFunction;

VarSize = ProblemSettings.VarSize;

VarMin = ProblemSettings.VarMin;

VarMax = ProblemSettings.VarMax;

global ICASettings;

beta = ICASettings.beta;

nEmp = numel(emp);

    for k = 1:nEmp

        for i = 1:emp(k).nCol

emp(k).Col(i).Position.m = emp(k).Col(i).Position.m…+beta*rand (VarSize.m).*(emp(k).Imp.Position.m- emp(k).Col(i).Position.m);

emp(k).Col(i).Position.m = max(emp(k).Col(i).Position.m,VarMin.m);

emp(k).Col(i).Position.m = min(emp(k).Col(i).Position.m,VarMax.m);

emp(k).Col(i).Position.sigma = emp(k).Col(i).Position.sigma…

+ beta*rand(VarSize.sigma).*(emp(k).Imp.Position.sigma-emp(k).Col(i).Position.sigma);

emp(k).Col(i).Position.sigma = max(emp(k).Col(i).Position.sigma,VarMin.sigma);

emp(k).Col(i).Position.sigma = min(emp(k).Col(i).Position.sigma,VarMax.sigma);

emp(k).Col(i).Position.w = emp(k).Col(i).Position.w…

+ beta*rand(VarSize.w).*(emp(k).Imp.Position.w-emp(k).Col(i).Position.w);

emp(k).Col(i).Position.w = max(emp(k).Col(i).Position.w,VarMin.w);

emp(k).Col(i).Position.w = min(emp(k).Col(i).Position.w,VarMax.w);

emp(k).Col(i).Cost emp(k).Col(i).Sol] = CostFunction(emp(k).Col(i).Position);

end

end

end

## Appendix C: DoRevolution ()

function emp = DoRevolution(emp)

global ProblemSettings;

    CostFunction = ProblemSettings.CostFunction;

    nVar = ProblemSettings.nVar;

    VarSize = ProblemSettings.VarSize;

    VarMin = ProblemSettings.VarMin;

    VarMax = ProblemSettings.VarMax;

global ICASettings;

    pRevolution = ICASettings.pRevolution;

    mu = ICASettings.mu;

    nmu = ceil(mu*nVar);

    SIGMA.m = 0.15*(VarMax.m-VarMin.m);

    SIGMA.sigma = 0.15*(VarMax.sigma-VarMin.sigma);

    SIGMA.w = 0.15*(VarMax.w-VarMin.w);

    nEmp = numel(emp);

    for k = 1:nEmp

    NewImp = emp(k).Imp;

      Q = randi([1 3]);

    jj = randsample(nVar,nmu)';

        switch Q

          case 1

NewPos.m = NewImp.Position.m + SIGMA.m*randn(VarSize.m);

NewImp.Position.m(jj) = NewPos.m(jj);

NewImp.Position.m = max(NewImp.Position.m,VarMin.m);

NewImp.Position.m = min(NewImp.Position.m,VarMax.m);

          case 2

NewPos.sigma = NewImp.Position.sigma + SIGMA.sigma*randn(VarSize.sigma);

NewImp.Position.sigma(jj) = NewPos.sigma(jj);

NewImp.Position.sigma = max(NewImp.Position.sigma,VarMin.sigma);

NewImp.Position.sigma = min(NewImp.Position.sigma,VarMax.sigma);

          case 3

NewPos.w = NewImp.Position.w + SIGMA.w*randn(VarSize.w);

NewImp.Position.w(jj) = NewPos.w(jj);

NewImp.Position.w = max(NewImp.Position.w,VarMin.w);

NewImp.Position.w = min(NewImp.Position.w,VarMax.w);

end

NewImp.Cost NewImp.Sol] = CostFunction(NewImp.Position);

if NewImp.Cost<emp(k).Imp.Cost

emp(k).Imp = NewImp;

       end

for i = 1:emp(k).nCol

        if rand< = pRevolution

Q = randi([1 3]);

jj = randsample(nVar,nmu)';

switch Q

            case 1

NewPos.m = emp(k).Col(i).Position.m + SIGMA.m*randn(VarSize.m); emp(k).Col(i).Position.m(jj) = NewPos.m(jj); emp(k).Col(i).Position.m = max(emp(k).Col(i).Position.m,VarMin.m); emp(k).Col(i).Position.m = min(emp(k).Col(i).Position.m,VarMax.m);

            case 2

NewPos.sigma = emp(k).Col(i).Position.sigma + SIGMA.sigma *randn (VarSize.sigma);

emp(k).Col(i).Position.sigma(jj) = NewPos.sigma(jj);

              emp(k).Col(i).Position.sigma = max(emp(k).Col(i).Position.sigma,VarMin.sigma);

              emp(k).Col(i).Position.sigma = min(emp(k).Col(i).Position.sigma,VarMax.sigma);

            case 3

NewPos.w = emp(k).Col(i).Position.w + SIGMA.w*randn(VarSize.w);

emp(k).Col(i).Position.w(jj) = NewPos.w(jj);

emp(k).Col(i).Position.w = max(emp(k).Col(i).Position.w,VarMin.w);

emp(k).Col(i).Position.w = min(emp(k).Col(i).Position.w,VarMax.w);

end

[emp(k).Col(i).Cost emp(k).Col(i).Sol] = CostFunction(emp(k).Col(i).Position);

          end

        end

      end

end

## Appendix D: InterEmpireCompetition ()

function emp = InterEmpireCompetition(emp)

if numel(emp) = = 1

         return;

    end

    global ICASettings;

    alpha = ICASettings.alpha;

    TotalCost = [emp.TotalCost];

    [~, WeakestEmpIndex] = max(TotalCost);

    WeakestEmp = emp(WeakestEmpIndex);

    P = exp(-alpha*TotalCost/max(TotalCost));

    P(WeakestEmpIndex) = 0;

    P = P/sum(P);

    if any(isnan(P))

      P(isnan(P)) = 0;

      if all(P = = 0)

          P(:) = 1;

      end

      P = P/sum(P);

    end

    if WeakestEmp.nCol>0

        [~, WeakestColIndex] = max([WeakestEmp.Col.Cost]);

        WeakestCol = WeakestEmp.Col(WeakestColIndex);

        WinnerEmpIndex = RouletteWheelSelection(P);

        WinnerEmp = emp(WinnerEmpIndex);

        WinnerEmp.Col(end+1) = WeakestCol;

        WinnerEmp.nCol = WinnerEmp.nCol+1;

        emp(WinnerEmpIndex) = WinnerEmp;

        WeakestEmp.Col(WeakestColIndex) = [];

        WeakestEmp.nCol = WeakestEmp.nCol-1;

        emp(WeakestEmpIndex) = WeakestEmp;

    end

    if WeakestEmp.nCol = = 0

        WinnerEmpIndex2 = RouletteWheelSelection(P);

        WinnerEmp2 = emp(WinnerEmpIndex2);

        WinnerEmp2.Col(end+1) = WeakestEmp.Imp;

        WinnerEmp2.nCol = WinnerEmp2.nCol+1;

        emp(WinnerEmpIndex2) = WinnerEmp2;

        emp(WeakestEmpIndex) = [];

    end

    end

## Appendix E: IntraEmpireCompetition ()

function emp = IntraEmpireCompetition(emp)

    nEmp = numel(emp);

      for k = 1:nEmp

        for i = 1:emp(k).nCol

           if emp(k).Col(i).Cost<emp(k).Imp.Cost

             imp = emp(k).Imp;

             col = emp(k).Col(i);

             emp(k).Imp = col;

             emp(k).Col(i) = imp;

           end

         end

       end

end

function [z sol] = MyCost(s,data)

    x = data.x;

    y = data.y;

    xmin = data.xmin;

    xmax = data.xmax;

    ws.w;

    ms.m;

    sigma = s.sigma;

    nKernel = numel(w);

      % nData = numel(x);

      % yhat = zeros(size(y));

      % for k = 1:nData

          % yhat(k) = 0;

          % for i = 1:nKernel

            % yhat(k) = yhat(k)+w(i)*exp(-0.5*((x(k)-m(i))/sigma(i))^2);

          % end

      % end

    xx = linspace(xmin,xmax,200);

    yy = zeros(size(xx));

    yhat = zeros(size(y));

    for i = 1:nKernel

      yhat = yhat+w(i)*exp(-0.5*((x-m(i))/sigma(i)).^2);

      yy = yy+w(i)*exp(-0.5*((xx-m(i))/sigma(i)).^2);

    end

    e = y-yhat;

    MSE = mean(e.^2);

    RMSE = sqrt(MSE);

    z = MSE;

    sol.x = x;

    sol.y = y;

    sol.xmin = xmin;

    sol.xmax = xmax;

    sol.yhat = yhat;

    sol.e = e;

    sol.MSE = MSE;

    sol.RMSE = RMSE;

    sol.xx = xx;

    sol.yy = yy;

end
